# Ionizing radiation, cerebrovascular disease, and consequent dementia: A review and proposed framework relevant to space radiation exposure

**DOI:** 10.3389/fphys.2022.1008640

**Published:** 2022-10-25

**Authors:** Kathleen B. Miller, Kaitlyn L. Mi, Gregory A. Nelson, Ryan B. Norman, Zarana S. Patel, Janice L. Huff

**Affiliations:** ^1^ National Institute of Aerospace, Hampton, VA, United States; ^2^ Brown University, Providence, RI, United States; ^3^ Department of Basic Sciences, Division of Biomedical Engineering Sciences, Loma Linda University, Loma Linda, CA, United States; ^4^ NASA Johnson Space Center, Houston, TX, United States; ^5^ KBR Inc., Houston, TX, United States; ^6^ NASA Langley Research Center, Hampton, VA, United States

**Keywords:** space radiation, cerebrovascular disease, stroke, dementia, vascular dementia, neurodegenerative disease, ionizing radiation, space travel

## Abstract

Space exploration requires the characterization and management or mitigation of a variety of human health risks. Exposure to space radiation is one of the main health concerns because it has the potential to increase the risk of cancer, cardiovascular disease, and both acute and late neurodegeneration. Space radiation-induced decrements to the vascular system may impact the risk for cerebrovascular disease and consequent dementia. These risks may be independent or synergistic with direct damage to central nervous system tissues. The purpose of this work is to review epidemiological and experimental data regarding the impact of low-to-moderate dose ionizing radiation on the central nervous system and the cerebrovascular system. A proposed framework outlines how space radiation-induced effects on the vasculature may increase risk for both cerebrovascular dysfunction and neural and cognitive adverse outcomes. The results of this work suggest that there are multiple processes by which ionizing radiation exposure may impact cerebrovascular function including increases in oxidative stress, neuroinflammation, endothelial cell dysfunction, arterial stiffening, atherosclerosis, and cerebral amyloid angiopathy. Cerebrovascular adverse outcomes may also promote neural and cognitive adverse outcomes. However, there are many gaps in both the human and preclinical evidence base regarding the long-term impact of ionizing radiation exposure on brain health due to heterogeneity in both exposures and outcomes. The unique composition of the space radiation environment makes the translation of the evidence base from terrestrial exposures to space exposures difficult. Additional investigation and understanding of the impact of low-to-moderate doses of ionizing radiation including high (H) atomic number (Z) and energy (E) (HZE) ions on the cerebrovascular system is needed. Furthermore, investigation of how decrements in vascular systems may contribute to development of neurodegenerative diseases in independent or synergistic pathways is important for protecting the long-term health of astronauts.

## 1 Introduction

Spaceflight and human presence outside of Earth’s magnetosphere pose several challenges for human health and performance. As astronauts embark on longer space flights with the goal of exploration beyond low-Earth orbit, they will be exposed to multiple environmental stressors. These include altered gravity fields, long periods of isolation and confinement, closed living and working quarters, stressful working conditions, large distances from Earth, loss of sleep, altered light-dark periods and circadian cues, and exposure to space radiation. Importantly, space radiation exposure remains one of the greatest risks to human health in space because of its potential for both acute and chronic health effects (Patel et al., 2020).

The health effects of terrestrial ionizing radiation exposure are well studied and documented for a wide range of ionizing radiation types and dosages, including Japanese atomic bomb survivors (Shimizu et al., 2010; Yamada et al., 2016; Little et al., 2020), those with therapeutic exposures (Johannesen et al., 2003; Darby et al., 2013; Domina, 2017) and those with occupational exposures (Boice et al., 2019; Hunter et al., 2015; dos Santos Silva et al., 2013). NASA has documented risks of exposure to the space radiation environment concerning cancer (Huff et al., 2016), central nervous system (CNS) decrements (Nelson G. A. et al., 2016) and cardiovascular and other degenerative tissue decrements (Patel et al., 2016). However, because of the relatively limited experience of humans in the space environment and the limited analysis in astronaut cohorts due to low sample size and power (Elgart et al., 2018), the evidence on the biological effects of space radiation is largely from terrestrial ionizing radiation exposures and preclinical experimental models of the space radiation environment. Importantly, the dose-rate and radiation quality effects from the space radiation environment differ from terrestrial exposures. Thus, for successful long-duration exploration missions in deep space, and for Lunar and Martian habitats, there is a need to identify potential physiological pathways by which the space radiation environment may impact astronaut health for radiation doses, dose-rates, and qualities relevant to those missions. These steps are necessary in order to inform risks and develop adequate countermeasures.

There is a growing concern about the risk of late occurring neurodegenerative diseases and cognitive decrements from exposure to the space radiation environment. Experimental results from simulated space radiation exposures in animal models have shown damages to CNS tissue including suppressed neurogenesis (Rola et al., 2004, 2005; Rivera et al., 2013; Cacao and Cucinotta, 2016), altered electrophysiological properties of the neurons (Vlkolinsky et al., 2010; Marty et al., 2014; Lee et al., 2017), changes in brain and neuronal structure including reduced dendritic branching (Parihar et al., 2015a; Carr et al., 2018; Howe et al., 2019), and increased chronic neuroinflammation (Poulose et al., 2011; Greene-Schloesser et al., 2012; Schnegg et al., 2012; Parihar et al., 2020) including changes in activation of microglia (Rosi, 2018; Allen et al., 2020; Rienecker et al., 2021). Ionizing radiation-induced CNS tissue decrements are associated with alterations in behavior and decreased cognitive function (Parihar et al., 2016; Cekanaviciute et al., 2018; Cucinotta and Cacao, 2019; Kiffer et al., 2019). Importantly, ionizing radiation exposure at doses relevant to NASA missions is known to increase risk for vascular damage, which can contribute to a variety of cardiovascular diseases including atherosclerosis, ischemic heart disease and stroke (Little. 2016; Patel et al., 2020). This evidence suggests that vascular factors, including damage to cerebral vessels and impairments in the blood brain barrier, may also be involved in increased risk for late neurodegeneration independently or synergistically with damage to the CNS tissues (Nelson A. R. et al., 2016). In addition, it has been noted that vascular tissue in the brain is particularly radiosensitive in humans (Nilsen et al., 2020) and in preclinical animal models (Reinhold and Hopewell, 1980; Yoshii and Phillips, 1982). Therefore, there is a need to identify the impact of the space radiation environment on the cerebrovascular system in order to understand the long-term risks for cerebrovascular diseases and consequent dementia.

The purpose of this work is to review epidemiological and experimental data regarding the impact of ionizing radiation on biological effects relevant to cerebrovascular disease and dementia with a focus on doses and qualities relevant to the space radiation environment. A mechanistic framework by which space radiation may lead to cerebrovascular, neural, and cognitive adverse outcomes is presented. The information presented is important for development of experimental and computational models necessary to estimate the risks for space radiation-induced late neurodegenerative diseases in astronauts following extended space missions.

## 2 The cerebrovascular system and cerebrovascular disease

### 2.1 Overview of the cerebrovascular system

**FIGURE 1:**
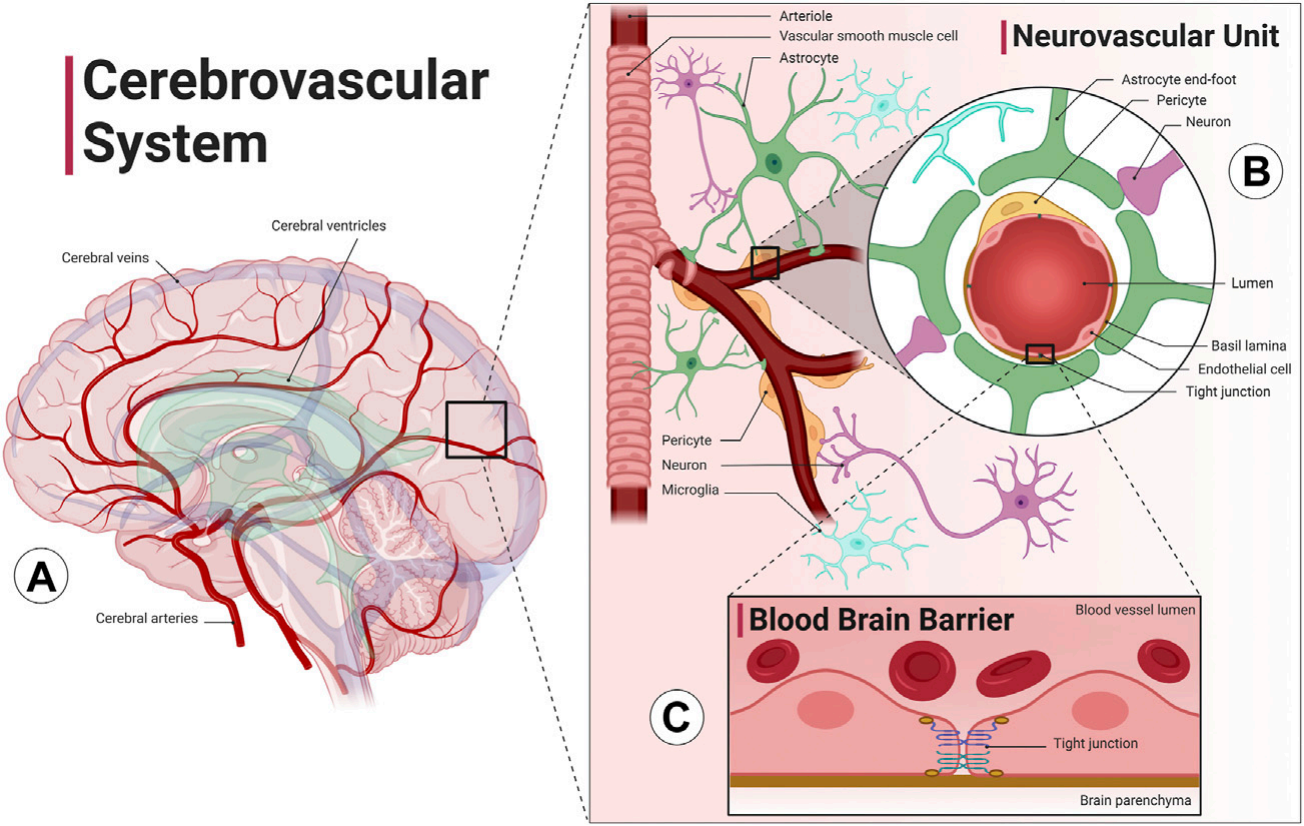
The cerebrovascular system. The cerebrovascular system is comprised of blood vessels that transport blood to and from the brain. **(A)** The cerebral arterial system (red) has four main arteries that supply the brain, the internal carotid arteries, which run along the neck and provide blood to the anterior cerebral cortex, and the vertebral arteries, which run along the spinal column and provide blood to the posterior cortex and the brainstem. The anterior and posterior cerebral circulation converge at the Circle of Willis, a group of blood vessels that anastomose at the base of the brain. The cerebral venous system (blue) includes superficial and deep veins that ultimately drain into two jugular veins at either side of the neck. Interconnected cavities called the cerebral ventricles (green) are filled with cerebrospinal fluid (CSF) and are involved with the brain’s unique waste management system, the glymphatic system. **(B)** The intracranial arteries branch and dive deep into the cortex, forming the intracerebral perforating arteries. Cerebral arterioles include vascular smooth muscle cells that allow the vessel to vasodilate or vasoconstrict in order to increase or decrease flow to the brain parenchyma. The neurovascular unit, consisting of blood vessels, neurons, astrocytes, pericytes and microglia cells, precisely regulates cerebral blood flow to match neuronal metabolic demand. **(C)** Cerebral capillaries lined with brain microvascular endothelial cells connected by tight junctions form the blood brain barrier, a specialized, semipermeable barrier that prevents entry of substances and pathogens into the brain as well as mediates molecular exchange and maintains the interstitial milieu. An additional barrier separates blood and CSF formed by choroid plexus epithelial cells (not shown). Image created with Biorender.com ^1^.

Relative to its size, the brain requires large amounts of blood flow due to its large metabolic demand. Specifically, cerebral blood flow utilizes about 15% of the total cardiac output despite being only about 2% of the total body weight (Xing et al., 2017). The cerebrovascular system (Figure 1A) is a highly sophisticated and organized vascular system responsible for maintaining adequate supply of oxygen and glucose in the brain, removing metabolic byproduct buildup, and temperature regulation. Neurons rely on the cerebrovascular system for operation, development, and survival; thus, the dense and organized network of vascular cells in the brain minimize diffusion distance between blood vessels and the brain parenchyma. The neurovascular unit (Figure 1B) consists of neurons, vascular cells (pericytes and endothelial cells), and glial cells (astrocytes, oligodendrocytes, and microglia). The neurovascular unit is the functional unit responsible for matching neuronal metabolism and cerebral blood flow (neurovascular coupling) (Kisler et al., 2017). The cerebrovascular system also includes the blood brain barrier, which provides^1^ the brain with a separate layer of protection against bacterial or viral infections (Figure 1C). The blood brain barrier is a semipermeable barrier that selectively transports various molecules critical to neuronal function while blocking many pathogens and peripheral immune cells. The blood brain barrier is formed by brain microvascular endothelial cells in brain capillaries that are connected by tight junctions and are surrounded by astrocyte end-feet (Ballabh et al., 2004). An additional barrier formed by choroid plexus epithelial cells separates blood and cerebrospinal fluid (CSF). CSF is involved in the brain’s unique waste management system, the glymphatic system, washing out solutes and metabolites using perivascular channels formed by astrocytes and glial cells. The glymphatic system also helps distribute other compounds such as glucose, lipids, amino acids and neurotransmitters and is largely active during sleep (Jessen et al., 2015). Ultimately, the health of all aspects of the cerebral circulation, including the large intracranial vessels, the intracerebral vessels, the neurovascular unit, and the blood brain barrier, are essential for optimal brain function.

### 2.2 Cerebrovascular diseases

Cerebrovascular diseases are conditions related to blood flow and blood vessels in the brain. Their presentations and outcomes are heterogeneous, ranging from no symptoms to severe symptoms such as permanent neurological damage or death. The most common type of cerebrovascular events are strokes, a major cause of death and disability worldwide (Donnan et al., 2008). Most strokes are classified as ischemic meaning they are caused by a blockage or narrowing of a blood vessel resulting in a loss in blood flow. Ischemic strokes can be caused by a thrombus occurring in the large extracranial or intracranial vessels or by an embolus of cardiac, aortic, pulmonary, or other origin traveling to the brain (Barthels and Das, 2020). By definition, strokes cause neurological dysfunction lasting over 24 h. Conversely, a transient ischemic attack is a transient episode of neurological dysfunction caused by ischemia but does not result in lasting neurological damage (Donnan et al., 2008). Regardless of the cause, ischemia can be particularly damaging in the brain as neural tissue can become necrotic in seconds to minutes (Lipton, 1999). Areas of ischemia-related necrotic tissue in the brain known as cerebral infarctions are generally classified by their size and location. For example, macroscopic infarctions are large infarctions in the cortical and subcortical regions, lacunar infarctions are small infarcts (2 mm–20 mm in diameter) in the basal ganglia, subcortical white matter or pons, and microinfarctions are lesions only visible by light microscopy (Grinberg and Thal, 2010). Strokes can also be classified as hemorrhagic meaning they result in excess blood or bleeding in the brain (Grysiewicz et al., 2008). There are two general types of hemorrhagic stroke: intracerebral hemorrhage with bleeding directly into the brain, and subarachnoid hemorrhage with bleeding near the pial surface of the brain into the cerebrospinal fluid. Common causes of hemorrhagic stroke include hypertension, trauma, or aneurysm (weakening and/or rupturing of a blood vessel). Ultimately, the treatment strategy and outcomes of stroke will range widely depending on the type, cause, location, and severity.

Cerebral small vessel disease is a general term for cerebrovascular dysfunction that affects the small cerebral arteries, arterioles, venules, and capillaries (Li et al., 2018). The criteria for clinical classification of cerebral small vessel disease varies; however, cerebral small vessel disease is usually identified on computed tomography (CT) or magnetic resonance imaging (MRI) brain scans based on the presence of subcortical infarcts, lacunar infarcts, white matter hyperintensities, and/or cerebral microbleeds (Wardlaw et al., 2013). The exact pathophysiology of cerebral small vessel disease is unknown, and the outcomes range from no symptoms to severe consequences such as stroke or dementia (Cannistraro et al., 2019). Unlike stroke, which is a cerebrovascular event that can last from seconds to minutes, cerebral small vessel disease can develop over decades (Smith et al., 2015) and is related to aging. Age-related increases in CT or MRI biomarkers of cerebral small vessel disease are commonly reported (Li et al., 2020). Those with cerebral small vessel disease may have cerebral hypoperfusion, impaired cerebral autoregulation, reduced neurovascular coupling, and increased blood brain barrier permeability. Cerebral small vessel disease has also been proposed as part of the pathophysiology of both vascular dementia and Alzheimer’s disease (Williams et al., 2019; Kim et al., 2020).

There are many potential factors that may contribute to the development of cerebrovascular diseases in both the large and small cerebral arteries. One of the main contributors is atherosclerosis, a buildup of plaque in the blood vessels. Atherosclerotic plaques are caused by the accumulation of lipids and fibrous elements prone to rupturing and causing a thrombus. In the context of stroke, atherosclerosis is the most common cause of local disease in the large extracranial vessels and large intracranial vessels (Marulanda-Londoño and Chaturvedi, 2016). The presence of atherosclerosis also creates an inflammatory environment which may have a systemic impact on cerebral vessels, as carotid atherosclerosis is associated with cerebral small vessel disease severity (Evans et al., 2021). Atherosclerosis can also occur in the cerebral microvessels, which are also prone to vascular stiffening from lipohyalinosis, a process where a penetrating vessel artery is blocked by lipids and fibroids and leads to wall thickening and thinning of the luminal diameter (Shindo et al., 2020).

Endothelial cell dysfunction and resulting inflammation are also common causes of cerebrovascular dysfunction in both large and small vessels. Endothelial dysfunction is a precursor to many vascular conditions including atherosclerosis, coronary artery disease, diabetes, and hypertension (Wang et al., 2018). Endothelial cells are critical for blood vessel function as they are responsible for regulating blood flow and forming the blood brain barrier. Healthy endothelial cells produce endothelium-derived nitric oxide (NO), a potent vasodilator. However, dysfunctional endothelial cells may have reduced NO bioavailability and difficulty regulating blood flow. There is a growing body of evidence suggesting that elevated oxidative stress, including production of reactive oxygen species and reactive nitrogen species, is a major cause of endothelial dysfunction in the cerebral circulation (Cahill-Smith and Li, 2014). Furthermore, since specialized brain microvascular endothelial cells create the blood brain barrier, dysfunctional endothelial cells may increase blood brain barrier permeability, leading to exposure of the neural cells to harmful environments (Ballabh et al., 2004).

Cerebral amyloid angiopathy (CAA) is also a potential mechanism contributing to the development of cerebrovascular diseases. CAA is a condition wherein beta amyloid peptides are deposited within small and medium sized cerebral blood vessels. CAA is age-dependent, and often occurs sporadically, with most patients 60 years of age or older. Therefore, the presence of CAA is not always associated with disease (Rosand et al., 2005). However, CAA can lead to increased risk of cerebral microbleeds, as well as incidence of both vascular dementia and Alzheimer’s disease (Pfeifer et al., 2002).

### 2.3 Connection between cerebrovascular disease and dementia

Dementia is an umbrella term that describes irreversible and progressive declines in cognitive function. The leading diagnosis of dementia is Alzheimer’s disease, characterized by the appearance of amyloid beta and tau pathologies in the brain, yet the exact pathophysiology of Alzheimer’s disease is still unclear. Vascular dementia, the second most common type of dementia, is characterized by a high load of vascular pathology in the brain (Gorelick et al., 2011). However, there is notable overlap in dementia pathologies, as most Alzheimer’s disease cases also exhibit a high load of vascular pathology (Santisteban and Iadecola, 2018) and Alzheimer’s disease patients may have high vascular risk (Lorius et al., 2015). Therefore, it has been suggested that vascular dysfunction is likely part of the etiology of multiple dementia types including Alzheimer’s disease, though it is unknown if vascular factors are additive or independent to Alzheimer’s disease pathologies. Vascular dysfunction contributing to dementia can manifest as overt cerebrovascular disease. History of stroke increases all causes of dementia risk by 70%, with recent strokes more than doubling the risk (Rist et al., 2013; Kuźma et al., 2018). Cerebral small vessel disease has also been reported to increase dementia risk (Liu et al., 2018; Cannistraro et al., 2019; Kim et al., 2020). Patients with dementia that have microvascular pathology have demonstrated significant loss of hippocampal neurons (Kril et al., 2002), including patients with hereditary cerebral small vessel disease (Yamamoto et al., 2021). Preclinical decrements in vascular function, i.e., changes that do not result in overt disease, may also contribute to dementia etiology. In a large cohort study of participants with late onset Alzheimer’s disease, analysis of plasma biomarkers suggested that vascular dysregulation was among the earliest in the cascade of events that were associated with disease progression (Iturria-Medina et al., 2016). In hypothetical disease models, cerebral microvascular dysfunction may lead to accumulation of amyloid pathologies in the brain and reduced ability to remove them, thereby accelerating the amyloid-dependent pathway of neurodegeneration (Kisler et al., 2017). Disruption of the blood brain barrier may also contribute to neurodegenerative pathologies and increase the prevalence of neuroinflammation and neurotoxins (Wu et al., 2005; Zlokovic, 2011; Heneka et al., 2015). In addition, global cerebral hypoperfusion can exacerbate neurodegenerative pathologies (Wolters et al., 2017). Because cerebrovascular dysfunction has been implicated in the pathophysiology of multiple dementia types including vascular dementia and Alzheimer’s disease, it is important to consider the health of the cerebrovascular system when determining dementia risk.

### 2.4 Summary of the cerebrovascular system and cerebrovascular diseases

The cerebrovascular system is a sophisticated vascular system essential for matching blood flow to the high metabolic demands of the brain. The main components of the cerebrovascular system include the intracranial vessels, intercranial vessels, the neurovascular unit, and the blood brain barrier. Cerebrovascular disease is a term used to describe general dysfunction of the cerebral circulation and can refer to a range of diseases including large or small vessels. Outcomes of cerebrovascular disease vary widely and can range from no symptoms to permanent neurological damage or death. General instigators of cerebrovascular dysfunction include atherosclerosis, arterial stiffening, CAA, and endothelial cell dysfunction, though exact mechanisms are unknown and will depend on a variety of environmental and genetic factors. Cerebrovascular dysfunction has been implicated in age-related neurodegenerative diseases such as dementia, including both vascular dementia and Alzheimer’s disease.

## 3 Evidence base describing the impact of ionizing radiation on the brain and cerebrovascular system–From preclinical models to humans

### 3.1 Overview of the space radiation environment

**FIGURE 2:**
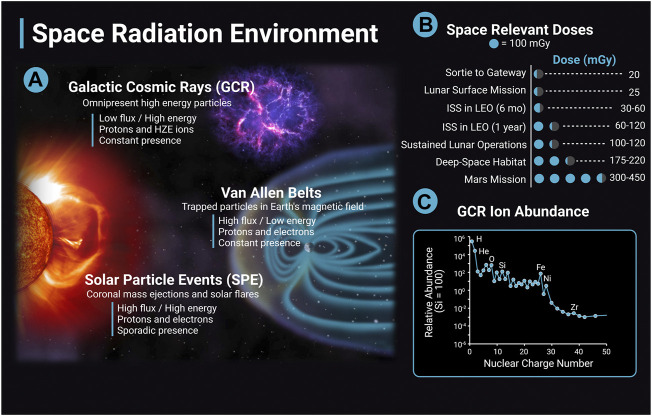
The space radiation environment. The space radiation environment consists of three main sources of radiation, solar particle events (SPE), Van Allen belts, and galactic cosmic rays (GCR). **(A)** GCR are high energy nuclei that originate from outside the solar system, possibly from supernovas, and are omnipresent. GCR have a low flux and high energy and consist largely of protons and high (H) atomic number (Z) and energy (E) (HZE) ions. The activity of GCR is anticorrelated with solar activity. SPEs are solar storms including solar flares and coronal mass ejections. They have high flux and high energy, consist mainly of protons and electrons, and the frequency of SPEs are cyclic depending on solar activity. A single SPE can last from hours to days. Van Allen Belts are trapped particles within Earth’s magnetic field that have high flux and low energy. Particles can be captured from solar events and GCR interactions with the Earth’s atmosphere. The inner belt is largely protons, and the outer belt is primarily electrons. **(B)** Space-relevant radiation doses in mGy for different missions are adapted from Simonsen et al. (2020). Dose estimates are during solar minimum, where GCR flux is at a maximum. **(C)** GCR ion abundance with ions relative to Si (Simpson et al., 2020). The y-axis data is in a log scale and is corrected for solar modulation. Image created with Biorender.com.

The following section will review the evidence base regarding the impact of ionizing radiation on cerebrovascular disease and dementia with a focus on low (<0.1 Gy) to moderate dose (0.1 Gy–0.5 Gy) ionizing radiation when available. The differences between the terrestrial radiation environment and the space radiation environment are important to note when evaluating the translatability of the preclinical evidence base and terrestrial exposures to astronaut risk. Astronauts can be exposed to ionizing radiation from three main sources: solar particle events (SPE), trapped radiation from Van Allen Belts, and galactic cosmic rays (GCR) (Figure 2). GCR are of particular concern, as they are an ominous, continuous presence in space and their potential for biological damage may be higher than terrestrial forms of ionizing radiation. Contrary to gamma- and X-rays with low-linear energy transfer (LET), GCRs include high-LET particle radiation of high (H) atomic number (Z) and energy (E) (HZE) ions that can produce densely ionized tracks as they transverse through biological tissues (Cucinotta and Durante, 2006) (Figure 2). Future missions in deep space will have a much greater expected ionizing radiation dose than previous International Space Station (ISS) or short lunar missions, and crews will experience greater GCR exposure. For example, ionizing radiation doses range from 30 mGy to 120 mGy for a 6 month to 12 months stay on the ISS; however, estimates for missions to Mars include ionizing radiation doses that are four to ten times greater (Norbury et al., 2016; Simonsen et al., 2020) (Figure 2). As a comparison, typical annual terrestrial exposures are less than approximately 5 mGy/year in the United States not including medical exposures (Metting, 2017).

### 3.2 Impact of ionizing radiation on the central nervous and cerebrovascular systems in humans and preclinical models: Focus on high-dose exposures

Central nervous system tissue has historically been considered “radioresistant” as clinical and neuroimaging assessments rarely noted brain tissue necrosis in patients irradiated with 50 Gy of ionizing radiation or below (Schultheiss et al., 1995). However, radiation-induced brain injury has been recognized for decades as a consequence of radiotherapy treatment (Meadows et al., 1975; Sheline et al., 1980; Ron et al., 1982) and better neuroimaging capabilities and improved experimental models have enhanced the understanding of radiation-induced brain injury at a variety of doses ranging from ones to tens and even hundreds of Gy (Baskar et al., 2012). There are three classifications of clinical radiation-induced brain injury: acute injury, which occurs immediately after irradiation and resolves within days; early-delayed injury, which occurs days to months following treatment and includes symptoms like headaches and short-term memory loss that are transient and reversible; and late-delayed injury, which occurs 6 months or later after irradiation and can include progressive and irreversible alterations in brain structure, vascular function, and cognition (Roman and Sperduto, 1995; Greene-Schloesser et al., 2012; Turnquist et al., 2020). Some estimates suggest that late-delayed injury occurs in over one-third of cranial radiotherapy patients (Johannesen et al., 2003). In addition, higher incidence of long-term cognitive impairment has been reported after therapeutic radiation exposures of the head and neck (Chen et al., 2015).

Mechanisms of late-delayed radiation-induced injury are complex and likely include multiple interacting pathological processes including neuroinflammatory pathology (Greene-Schloesser et al., 2012, 2013) death of neural progenitor cells and inhibition of neurogenesis (Rola et al., 2004). There is also evidence that damage to the vascular endothelium may be a primary, or a significant contributing mechanism in the development of late radiation-induced CNS injury (Schultheiss et al., 1995; Hopewell, 1998). For example, single doses of 20 Gy–25 Gy of X-rays to the brain in rats caused endothelial cell damage that was apparent prior to development of necrosis or other changes in white matter (Calvo et al., 1988; Reinhold et al., 1990). Further, rats that developed vascular damage but did not develop necrosis, still demonstrated cognitive deficits, though the impairments were significantly less than animals that developed necrosis in certain domains (Hodges et al., 1998). In 50% of rats irradiated with a single dose of 25 Gy of X-rays, radiation-induced brain necrosis was present 65 weeks later. However, in rats given a radioprotector restricted to the vasculature, radiation-induced necrosis was only present in 10% of rats (Lyubimova and Hopewell, 2004). Also, using boron neutron capture therapy, the majority of direct damage to the spinal cord from neutrons is limited to the endothelial cell lining (Coderre and Morris, 1999; Coderre et al., 2006). The exact pathophysiology is not clear, though endothelial cell dysfunction may be related to upregulation of endothelial adhesion molecules, which has been shown after large doses of X-ray radiation in human aortic endothelial cells (Khaled et al., 2012) and mice (Kyrkanides et al., 1999; Yuan et al., 2005), or endothelial cell apoptosis *via* ceramide after activation of the sphingomyelinase pathway, which has been shown at very high doses (50 + Gy) (Peña et al., 2000; Li et al., 2003; Wong and Van der Kogel, 2004).

High doses of cranial ionizing radiation may also cause changes in blood vessel volume and density. For example, mice subject to 9 Gy of head-only X-ray irradiation demonstrated reduced mean vessel volume by 34% 2 days post exposure and rats subject to 10 Gy of head-only irradiation demonstrated a threefold drop in capillary density 1 month after exposure compared with controls (Craver et al., 2016). Furthermore, mice given 40 Gy of X-rays over 4 weeks developed 30% vascular rarefaction by 10 weeks, though vessel density returned to baseline values by 20 weeks (Brown et al., 2005). Mice subject to 36 Gy of whole brain radiation demonstrated profound capillary rarefaction in the hippocampus 1 month post exposure despite local tissue hypoxia. Yet, treatment with systemic hypoxia for 1 month caused complete restoration of the tissue density (Warrington et al., 2011). Vessel perfusion and oxygenation was also reduced in the brains of mice irradiated with a single dose of 20 Gy of photons with a degree of recovery occurring by 60 days (Ansari et al., 2007).

Compared with controls who had a prior single thoracic exposure of 10 Gy, non-human primates exposed to high doses of fractionated whole brain irradiation at 40 Gy demonstrated cerebrovascular and white matter lesions and upregulation of genes related to cerebrovascular remodeling and neuroinflammation especially in the white matter (Andrews et al., 2017). Long-term radiation induced cerebrovascular impairment has also been observed in non-human primates exposed to much lower doses. In a cohort of 120 animals administered a single-dose of total body irradiation between 1.14 and 8.5 Gy, 13% of animals developed brain lesions indicative of cerebrovascular damage compared with 0% of the non-irradiated controls. In the animals that had at least one brain lesion present, 7 animals developed new lesions during the surveillance period (3.7–11.3 years post-irradiation) (Andrews et al., 2020). Whether similar changes occur in non-human primate brains following low-to-moderate dose ionizing radiation exposure is not known.

Taken together, ionizing radiation can have long-term impacts on both brain structure and function at doses relevant to therapeutic applications in humans and in animal models. It is also possible that ionizing radiation-induced damage to the vascular endothelium as well as decreased vascular density and impairments in angiogenesis may play a primary or at least contributing role in late CNS decrements from high dose exposures (Warrington et al., 2013). However, it is not clear how these results regarding ionizing radiation-induced brain injury from therapeutic exposures at doses from ones to tens or hundreds of Gy translate to astronauts experiencing lower doses, different dose-rates, and different types of radiation.

### 3.3 Impact of ionizing radiation on the central nervous system in preclinical models: Focus on high LET exposures at low-to-moderate doses

Along with the growing body of evidence suggesting radiation-induced brain injury can occur in the dose ranges relevant to therapeutic exposures (ones to tens and even hundreds of Gy), exposure to low (<0.1 Gy) or moderate (0.1 Gy–0.5 Gy) doses of high LET ionizing radiation may also cause alterations to brain structure and function, although most of the data comes from preclinical studies. Data from studies conducted using similar doses of low LET radiation are lacking. Multiple experimental studies of mice irradiated with <0.5 Gy of proton or HZE ions have shown acute cognitive decrements in behavior, attention and memory (Parihar et al., 2016; Cucinotta and Cacao, 2019). Cognitive decrements are evident in mice irradiated with single species of ionizing radiation, protons or HZE ions (Davis et al., 2015; Britten et al., 2018; Parihar et al., 2018; Howe et al., 2019) as well as combined ion exposures (Raber et al., 2016, 2019; Kiffer et al., 2018, 2020). For a review of studies evaluating animal behavioral effects see Kiffer et al. (2019) and figure 1 from Cekanaviciute et al. (2018).

Consistent with cognitive deficits, numerous structural and functional deficits of CNS tissues have been reported in animals exposed to experimental simulation of the space radiation environment. For example, mice irradiated with a five beam mixed-ion GCR simulator at a low dose (30 cGy) demonstrated altered inhibitory neuronal signaling, disrupted hippocampal network activity and decrements in learning, memory and anxiety responses compared with non-irradiated mice suggesting that cognitive decrements are related to structural and functional changes in CNS tissues (Klein et al., 2021). Multiple studies of low-to-moderate dose exposure to HZE ions have demonstrated reduced hippocampal neurogenesis in mice (Rola et al., 2004, 2005, 2008; Rivera et al., 2013; Cacao and Cucinotta, 2016; Sweet et al., 2016; Whoolery et al., 2017). There may be additional complex, dynamic, time dependent effects of HZE exposure on neurogenesis. Two months post exposure of 10 cGy of ^56^Fe ions, neurogenesis was impaired. However, levels of adult-born neurons rebounded significantly above control levels after 12 months (Miry et al., 2021). Other structural tissue deficits in experimental rodent models irradiated with HZE ions include reduced number of dendritic spines and synaptic degeneration (Parihar et al., 2015a; Krukowski et al., 2018a; Carr et al., 2018; Kiffer et al., 2020). For example, 8 weeks after exposure to low doses of ^16^O and ^48^Ti ions, dendrites in the medial prefrontal cortex showed an approximate 30% reduction in length and branching (Parihar et al., 2015a), and ionizing radiation-induced spine loss may be sex-specific (Hinkle et al., 2019). Changes in the electrophysiological properties of neurons and synapses have also been reported with specific impairment of inhibitory activity (Vlkolinsky et al., 2010; Marty et al., 2014; Lee et al., 2017). Furthermore, gene expression changes in the brain, including a down-modulation of genes involved in neural signaling activity, are evident after low-dose ionizing radiation exposure (Yin et al., 2003) and in combination with hind-limb unloading (Overbey et al., 2019).

In addition to an impact on the structure and function of neurons, it is possible that exposure to ionizing radiation may increase neuroinflammation and subsequent microglia activation. For a review of neuronal damage and neural inflammation in animal models exposed to HZE ions and simulated GCR exposure, see figure 2 from Cekanaviciute et al. (2018). Multiple studies have suggested that cranial irradiation significantly increases neuroinflammation, especially at high doses (Moravan et al., 2011; Schnegg et al., 2012; York et al., 2012; Morganti et al., 2014). However, components of the neuroinflammatory cascade such as activation of microglia and increased levels of reactive oxygen species have been observed at doses of HZE ions much lower than 1 Gy. Notably, microglial cells, which are responsible for regulating the immune health of the neurons and synapses, are activated in response to low doses of helium ion exposure (Krukowski et al., 2018a, 2018b; Parihar et al., 2018; Rosi, 2018; Allen et al., 2020; Rienecker et al., 2021). In fact, a temporary depletion of microglial populations restored cognitive and behavioral deficits in mice that occurred as a result of low doses of helium ion exposure, showing encouraging results as a potential countermeasure (Rosi, 2018; Allen et al., 2020; Rienecker et al., 2021).

Increased levels of oxidative stress, including production of both reactive oxygen species and reactive nitrogen species, have also been reported in mice and human neural stem cells irradiated with protons and HZE ions (Limoli et al., 2007; Acharya et al., 2010; Tseng et al., 2013). Importantly, increased oxidative stress and redox imbalance is likely part of the pathogenesis of ionizing radiation-induced brain effects. Both transgenic mice with enhanced hydrogen peroxide and superoxide detoxification capacity (Liao et al., 2013; Parihar et al., 2015b), as well as treatment with an antioxidant alpha-lipoic acid (Manda et al., 2008; Villasana et al., 2013), suppressed radiation-induced impairments in neurogenesis and cognition. Amifostine, a free radical scavenger, has also been shown to protect against decrements in novel object recognition tests two to 3 months after 500 mGy of five ion radiation exposure (protons, ^28^Si, ^4^He, ^16^O, and ^56^Fe) in male mice (Boutros et al., 2021). Interestingly, in transgenic mice with a superoxide dismutase knockout, radiation-induced impairment of neurogenesis was suppressed, suggesting accumulation of superoxide may act as a primer for protection against ionizing radiation related reductions in neurogenesis (Rola et al., 2007; Fishman et al., 2009; Fike, 2011). Therefore, oxidative stress and redox homeostasis may play an important role in ionizing radiation-induced effects on neurogenesis and cognitive function.

To summarize, studies of animal models suggest that exposure to ionizing radiation, especially by HZE ions, may elicit deficits in cognitive function, as well as structural and functional deficits to the neurons and glial cells. Increased neuroinflammation and oxidative stress are likely part of the pathophysiological process of radiation-induced injury to CNS tissue, though the time course of the neuroinflammatory cascade, relative doses of low-LET *versus* high-LET radiation required to elicit these responses, as well as the long-term impact of ionizing radiation-induced neuroinflammation are still unknown. Furthermore, there are still many questions that remain when translating cognitive assays from preclinical models to humans.

### 3.4 Impact of ionizing radiation on neurodegenerative diseases in preclinical models: Focus on high LET exposures at low-to-moderate doses

Few studies have evaluated the impact of exposure to HZE ions found in the space radiation environment on long-term risk for neurodegenerative diseases in experimental models. In male Gottingen minipigs, 1.79 Gy of Cobalt (^60^Co) gamma-rays elicited lower levels of tau and amyloid pathology in specific brain areas susceptible to Alzheimer’s pathology verses sham controls (Iacono et al., 2021). However, this was not a high-LET exposure, and rodent models have shown mixed results related to radiation-induced changes in neurodegenerative pathology. In transgenic mouse models that overexpress the human amyloid precursor protein, ^56^Fe ion irradiation of 1 Gy–4 Gy at 1 GeV/u resulted in long-term age-related behavioral abnormalities as well as deficits in synaptic transmission consistent with Alzheimer’s disease related neurological deficits (Vlkolinsky et al., 2010). In a mouse model of Alzheimer’s disease expressing chimeric mouse/human amyloid precursor protein and mutant human presenilin-1 (APP/PSI), 10 cGy and 100 cGy ^56^Fe ion radiation resulted in decreased cognitive abilities 6 months later (Cherry et al., 2012). Male mice had accelerated amyloid beta pathology consistent with Alzheimer’s disease. In addition, mice irradiated with 100 cGy ^56^Fe ions showed evidence of endothelial activation, suggesting reductions in blood brain barrier integrity (Cherry et al., 2012). Also, in APP/PSI mice, whole-body dose of 10 cGy or 50 cGy ^56^Fe ions at 1 GeV/u resulted in dose-dependent changes in cognitive function including locomotor activity, contextual fear conditioning, grip strength and motor learning one and a half months later (Liu et al., 2019). There were sex and phenotype specific changes in amyloid beta pathology and microhemorrhages, including a beneficial impact of radiation on cerebral amyloid beta levels and microglia on female transgenic mice (Liu et al., 2019; Schroeder et al., 2021). However, in a follow-up study of a triple transgenic mouse model of Alzheimer’s disease expressing both amyloid and tau pathology (3xTg mice), ^28^Si or ^56^Fe ion irradiation at either 10 cGy or 100 cGy did not alter either amyloid or tau pathology (Owlett et al., 2020). Therefore, some studies of transgenic mouse models of Alzheimer’s disease irradiated with HZE ions demonstrate an increase in pathologies related to Alzheimer’s disease progression but the dose, time since exposure, and sex effects are complex. Some studies suggest the possibility of protective effects of low-dose, low-LET radiation on Alzheimer’s disease pathologies, particularly in female animals (Liu et al., 2019; Iacono et al., 2021; Schroeder et al., 2021). There is limited data regarding long-term multiple exposures from mixed ions. Also, it is unclear how the transgenic mouse models that develop Alzheimer’s disease pathology may translate to long-term deficits in cognitive function and development of dementia in humans.

### 3.5 Risk for dementia and neurodegenerative diseases in humans exposed to low-to-moderate doses of ionizing radiation

As described in Section 3.2, higher incidence of long-term cognitive impairment has been reported after therapeutic radiation exposures of the head and neck in adults (Chen et al., 2015) and in pediatric patients (Mulhern et al., 2004; De Ruiter et al., 2013). However, the effect of low-to-moderate doses of radiation from therapeutic exposures on late neurodegenerative diseases are less clear. In pediatric patients, a systematic review found limited evidence for an association between low-dose ionizing radiation and late neurodevelopmental effects. However, the authors noted that heterogeneity between exposures and outcome measures made comparisons between cohorts difficult (Pasqual et al., 2020, 2021). Recent clinical trials are investigating the potential therapeutic effect of ionizing radiation on Alzheimer’s disease pathology but results from such clinical trials in humans have yet to be reported (Chung et al., 2021; Jebelli et al., 2022). The evidence for ionizing radiation-induced late neurodegenerative disorders from the Japanese Atomic Bomb Survivors as well as other cohorts with accidental exposures is also mixed. Analysis of a cohort of Japanese Atomic Bomb Survivors as part of the Adult Health Study did not show any radiation related increases in mortality from dementia in initial cohort studies (Yamada et al., 1999) or follow up studies (Yamada et al., 2009, 2016). However, recent reports from Chernobyl catastrophe cleanup workers and liquidators suggest that there may be an impact of low-dose ionizing radiation on the development of cognitive impairment. For example, in 326 Ukrainian Chernobyl cleanup workers, those with doses above 100 mSv had a higher prevalence of cognitive deficits compared with groups with lower or no exposure. The most severe deficits were seen in those with doses above 500 mGy (Bazyka et al., 2015). Follow-up studies of the Ukrainian Chernobyl cleanup workers also suggest a dose-dependent increase in cognitive impairment from radiation exposure (Loganovsky et al., 2018, 2020). However, in Estonian Chernobyl cleanup workers, there was no difference in morbidity for mental disorders in exposed compared with unexposed cohorts (Rahu et al., 2014).

A recent review and meta-analysis of 16 studies of cohorts of individuals occupationally exposed to ionizing radiation (nuclear workers and uranium miners, nuclear weapons test participants, and medical workers) reported a standardized mortality ratio of 0.86 (CI: 0.79–0.93) for mortality of diseases of the nervous system compared with general population as the reference (Lopes et al., 2022). However, the overall excess relative risk (ERR) at 100 mGy of Parkinson’s disease mortality and morbidity from four studies of nuclear industry workers and medical workers was 0.11 (CI: 0.06, 0.16) (Lopes et al., 2022). In participants of the INWORKS study, which includes over 300,000 nuclear workers from France, the United Kingdom and the United States, there was a significant ERR with 90% confidence intervals in mortality from mental disorders (ERR/Sv = 1.30, 90% CI: 0.23, 2.72). In addition, over 50% of the deaths from mental disorders were related to dementia (Gillies et al., 2017). Furthermore, in a study of over 20,000 Russian Mayak workers, there was a possible dose-dependent increase in the incidence of Parkinson’s disease (ERR/Gy = 1.02, 95% CI: 0.59, 1.63) (Azizova et al., 2020). Yet, individual cohorts of workers in the United States occupationally exposed to ionizing radiation, including workers at the Los Alamos National Laboratory and medical radiation workers, did not report any ionizing radiation-related increases in mortality from dementia, Alzheimer’s disease, Parkinson’s disease and other motor neuron diseases (Boice et al., 2021a; 2021b). However, additional analysis including the evaluation of combined cohorts of occupationally exposed individuals with updated dosimetry that will assess the ionizing radiation-related risks for neurodegenerative diseases including dementia and Parkinson’s disease are forthcoming (Boice et al., 2021c).

Therefore, though some studies suggest low-to-moderate doses of ionizing radiation may impact risk for late neurogenerative diseases, the evidence is limited, and combination of cohorts is difficult due to the heterogeneity between exposures and outcome measures. Many studies group multiple neurodegenerative diseases and dementia into the category of mental disorders and do not stratify by disease. Furthermore, many only report mortalities and not incidence or morbidity. Finally, it is possible that cohorts compared against the general population for a standard mortality ratio exhibit a large confounding influence of the healthy worker effect. As the populations of exposed workers continue to age, there is more research necessary to understand how low-to-moderate doses of ionizing radiation may impact risk for late neurodegenerative diseases including dementia.

To summarize, evidence from occupationally and accidentally exposed cohorts and experimental models suggests a potential connection between ionizing radiation at low-to-moderate doses and future risk for neurodegenerative diseases, though evidence is limited. More research is needed to understand how this risk may translate to astronauts exposed to the space radiation environment. There are many heterogeneities in exposures and outcomes in the epidemiological cohorts. Similarly, transgenic mouse models of Alzheimer’s disease show heightened neurodegenerative pathologies in response to exposure to HZE ions, but responses may be varied in individual animals and it is unclear how these pathologies may translate to humans.

### 3.6 Impact of ionizing radiation on the cerebrovascular system in preclinical models: Focus on low-to-moderate doses

Evidence from both low and high-LET experiments on the large central blood vessels like the aorta, as well as endothelial cell models, suggest that ionizing radiation-induced vascular stiffness, endothelial cell dysfunction, and accelerated progression of atherosclerosis are likely part of the pathogenic processes of radiation-induced vascular dysfunction (Takahashi et al., 2003; Grabham et al., 2011; Soucy et al., 2011; Yu et al., 2011; Grabham et al., 2013; Beck et al., 2014; White et al., 2015; Wang et al., 2016; Baselet et al., 2019; Mitchell et al., 2019; Wuu et al., 2020; Meerman et al., 2021) albeit many of the studies are of higher radiation doses than are relevant for NASA missions. Emerging evidence suggests that exposures of low-LET ionizing radiation under 0.5 Gy may also increase long-term risk for cardiovascular disease, and this has been the topic of multiple review articles (Kreuzer et al., 2015; Baselet et al., 2016; Tapio et al., 2021). Despite the evidence in the general vascular system, the impact of ionizing radiation on the blood vessels in the brain is less clear at low-to-moderate doses relevant for NASA missions. Low-dose and low-LET irradiation may cause alterations in neurovascular remodeling and impairments in the blood brain barrier. For example, in mice irradiated with low-dose gamma radiation using a cobalt-57 plate (0.01 cGy/h for a total dose of 0.04 Gy) combined with hindlimb unloading to simulate microgravity, there was increased expression of aquaporin-4 after 9 months, suggesting reduced blood brain barrier integrity (Bellone et al., 2016). Increased aquaporin-4 and reduced tight junction protein expressions were also observed in the brain retinal barrier of mice after whole-body proton irradiation of 0.5 Gy of either single or fractionated doses indicating a radiation-induced reduction in blood retinal barrier integrity (Mao et al., 2021). However, the impact of ionizing radiation on the blood brain barrier may be time dependent. For example, cranial X-ray doses of 0.1, 2, 10 Gy increased brain vessel permeability approximately two-fold in both the cerebrum and cerebellum 1 week after irradiation. But, effects were fully recovered by 26 weeks (Sándor et al., 2014).

Regarding high-LET radiation, in cultured human brain microvascular endothelial cells, irradiation with 10 cGy–75 cGy of ^56^Fe ions resulted in endothelial cell barrier dysfunction (Grabham et al., 2013; Sharma et al., 2014). In mice, there was a 34% and 29% loss of CA1 hippocampal microvessels 12 months post irradiation from 0.5 Gy to 2 Gy of ^56^Fe ions respectively. However, mice irradiated with 4 Gy ^56^Fe ions showed similar levels of microvessel cell density as control mice (Mao et al., 2010), which suggests that there may be a “U-shaped” dose response to high-LET radiation, possibly due to early initial rapid losses and repopulation of endothelial cells at the 4 Gy dose. Furthermore, there was no effect 12 months post any level of ^56^Fe ion irradiation on the endothelial cell population in the dentate gyrus of the hippocampus, suggesting there may also be regional susceptibility depending on the vascular topology of the brain region being irradiated (Mao et al., 2010).

In summary, multiple studies have suggested that exposure to both high and low-LET radiation can cause ionizing radiation-induced vascular damage, particularly endothelial dysfunction in the large central arteries. However, how ionizing radiation impacts the blood vessels in the brain specifically is less clear. At high doses, irreversible and persistent damage to the endothelial cells in the brain is apparent. At low-to-moderate doses, ionizing radiation may impact the endothelial cells and reduce blood brain barrier integrity as well as reduce microvascular density though outcomes depend on dose, LET, time and specific brain region. More research is needed regarding the impact of low-to-moderate dose ionizing radiation, especially comparisons between HZE ions and low-LET gamma rays, on the cerebrovascular system. Furthermore, additional studies may address how radiation-induced vascular decrements may impact CNS decrements, and if they are operating in independent or synergistic pathways.

### 3.7 Risk for cerebrovascular diseases in humans exposed to low-to-moderate doses of ionizing radiation

Vascular dysfunction and increased risk for cardiovascular disease in response to ionizing radiation has been reported from therapeutic exposures (Swerdlow et al., 2007; Mulrooney et al., 2009; Darby et al., 2013; Wang et al., 2019; Belzile-Dugas and Eisenberg, 2021), accidental exposures (Shimizu et al., 2010) or occupational exposures (Little et al., 2012; Little, 2016). In addition to cardiovascular diseases, increased morbidity and mortality from cerebrovascular diseases has also been a growing concern for those occupationally and accidentally exposed to low-to-moderate doses of ionizing radiation (Wakeford, 2022). However, not all studies report a significant ERR for ionizing radiation induced cerebrovascular disease (Table 1). An analysis of 10,339 Atomic bomb survivors in Hiroshima and Nagasaki, Japan in the Adult Health Study during 1958–1998 reported no significant ERR for cerebrovascular disease morbidity (Yamada et al., 2004). Yet, later analyses of an expanded cohort of the Japanese Atomic Bomb Survivors from 1950 to 2003 have suggested that doses above 0.5 Gy–0.75 Gy were sufficient to elicit an increase in stroke mortality using both linear no threshold (Shimizu et al., 2010) and multi-model inference ERR models (Schöllnberger et al., 2018). The type of stroke may also have an impact, as a prospective follow up study from 1980 to 2003 of Atomic Bomb Survivors in Hiroshima and Nagasaki observed no dose related increases in ischemic stroke incidence, however risk of hemorrhagic stroke increased with ionizing radiation exposure in both men at any dose level and in women above a threshold of 1.3 Gy (Takahashi et al., 2012). Data from other cohorts, or aggregation of multiple cohorts, suggest that there may be an effect at low-to-moderate doses. In a recent meta-regression analysis of combined cohorts including Japanese Atomic Bomb Survivors, occupationally exposed workers, environmentally exposed groups and therapeutically and diagnostically exposed groups, there was a significant ERR for cerebrovascular disease (ERR/Gy = 0.24, 95% CI: 0.06, 0.41), which was higher for low dose-rate exposure (ERR/Gy = 0.31, 95% CI: 0.08, 0.54) (Little, 2016). Another meta-analysis also reported an overall ERR at 100 mGy for mortality (ERR at 100 mGy = 0.01, 95% CI: 0.00, 0.02) and morbidity (ERR at 100 mGy = 0.04, 95% CI: 0.03, 0.05) from cerebrovascular diseases (Lopes et al., 2022). The INWORKS study reported a significant ERR for mortality with 90% confidence intervals due to cerebrovascular disease (ERR/Sv = 0.50, 90% CI: 0.12, 0.94) (Gillies et al., 2017). Furthermore, Chernobyl emergency workers and liquidators also had a positive ERR for cerebrovascular disease morbidity (Ivanov et al., 2006; Kashcheev et al., 2016). In 198 Ukrainian Chernobyl Catastrophe Liquidators, there was a significant increased relative risk (RR) of both acute cerebrovascular disorders (RR = 1.40, 95% CI: 1.3, 1.5) and chronic cerebrovascular disorders (RR = 1.23, 95% CI: 1.0, 1.5) in those exposed to >50 mSv compared to internal controls who were exposed to doses <50 mSv (Loganovsky et al., 2020). There was also a positive and significant ERR for cerebrovascular disease mortality in 166,812 nuclear workers from the United Kingdom National Registry for Radiation Workers (ERR/Sv = 0.57, 95% CI: 0.00, 1.31) with increased cerebrovascular disease mortality rates observed after doses as low as 10–20 mSv, though the analysis was not adjusted for cofounding factors such as socioeconomic status (Hinksman et al., 2022).

**TABLE 1:** Summary of epidemiological evidence describing impact of ionizing radiation on risk for cerebrovascular disease mortality or morbidity.

Reference	Title of Study	Population	Exposure Type	Dose Ranges	Endpoint / ICD Codes	Primary Results Related to Cerebrovascular Disease
**Meta-Analysis**						
Little (2016) ^†^	Radiation and Circulatory Diseases	Meta-analysis of therapeutically exposed groups, diagnostically exposed groups, Japanese Atomic Bomb Survivors, occupationally exposed groups, and environmentally exposed groups.	Multiple exposure types.	Multiple.	Cerebrovascular disease morbidity and mortality. ICD 10 codes: 160-169.	ERR/Gy = 0.236, (95% Confidence interval (CI): 0.062, 0.410) including Mayak morbidity data. ERR/Gy = 0.154, (95% CI: 0.000, 0.307) including Mayak mortality data.
**Japanese Atomic Bomb Survivors**						
Yamada et al. (2004)*	Noncancer Disease Incidence in Atomic Bomb Survivors	10,339 Atomic bomb survivors in Hiroshima and Nagasaki, Japan in the Adult Health Study (AHS) during 1958–1998.	Atomic bomb radiation.	Mean dose = 0.1 Gy, range = 0 to 4 Gy.	Cerebrovascular disease morbidity. ICD 9 codes: 430, 431,433,434,436.	ERR/Gy = 0.07, (95% CI: –0.08, 0.24) using morbidity data.
Shimizu et al. (2010)	Radiation Exposure and Circulatory Disease Risk: Hiroshima and Nagasaki Atomic Bomb Survivor Data, 1950-2003	86,611 Atomic bomb survivors in Hiroshima and Nagasaki, Japan from the Life Span Study.	Atomic bomb radiation.	Mean dose = 0.1 Gy, range = 0 to 4 Gy.	Cerebrovascular disease mortality. All ICD codes converted to ICD 9. ICD 9 codes: 430-438.	ERR/Gy = 0.12, (95% CI: 0.05, 0.19).
Takahashi et al. (2012)*	A Prospective Follow-up Study of the Association of Radiation Exposure with Fatal and Non-fatal Stroke Among Atomic Bomb Survivors in Hiroshima and Nagasaki (1980 - 2003)	9,515 Atomic bomb survivors from the Adult Health Study in Hiroshima and Nagasaki, Japan.	Atomic bomb radiation.	Men mean dose = 0.41±0.62 Gy. Women mean dose = 0.36±0.55 Gy.	Cerebrovascular disease morbidity. ICD 7 codes: 330 - 332, 334, 352 and 435, ICD 8 codes: 333, 430 - 434, 436 and 438, ICD 9 codes: 430, 431 and 433-438, ICD 10 codes: G45, I60, I61, I63- 66 and I69 (exclude I698). All data (health examinations, death certificates, and autopsy reports) were evaluated. Stroke subtypes (ischemic and hemorrhagic) were based on clinical features, neuroimaging, and other laboratory criteria if available.	Radiation dose was unrelated to ischemic stroke risk. For hemorrhagic stroke, women with exposures above 1.3 Gy, relative hazard = 1.4, (95% CI: 0.6 to 3.7), women with exposures above 2.2 Gy, relative hazard = 3.5 (95% CI: 1.4, 9.0). Men with exposures above 1 Gy relative hazard = 1.7 (95% CI: 0.7, 4.1). Men with exposures above 2 Gy relative hazard = 2.5 (95% CI: 0.8, 7.3). No apparent threshold. All using morbidity data.
Schöllnberger et al. (2018)	Dose-responses for Mortality from Cerebrovascular and Heart Diseases in Atomic Bomb Survivors: 1950–2003	86,611 Atomic bomb survivors in Hiroshima and Nagasaki, Japan from the Life Span Study.	Atomic bomb radiation.	Mean dose = 0.1 Gy, range = 0 to 4 Gy.	Cerebrovascular disease mortality. ICD 9 codes: 430-438.	Multi-model inference indicated sublinear dose-response mortalities from cerebrovascular diseases at low and medium doses (0-1.4 Gy) however these were not statistically significant.
**Occupationally Exposed Workers**						
Vrijheid et al. (2007)	Mortality from Diseases other than Cancer Following Low Doses of Ionizing Radiation: Results from the 15-Country Study of Nuclear Industry Workers	275,312 workers from 15 different countries with adequate information on socioeconomic status. 11,255 workers had died of non-cancer diseases.	Individuals engaged in the production of nuclear power, the manufacture of nuclear weapons, the enrichment and processing of nuclear fuel, the production of radioisotopes or reactor or weapons research; uranium mining is not included.	Mean dose = 0.0207 Gy, range = 0.0 to 0.5 Gy.	Cerebrovascular disease mortality. ICD 10 codes: 160-169.	ERR/Gy = 0.88, (95% CI: -0.67, 3.16).
Lane (2010)	Mortality (1950-1999) and cancer Incidence (1969-1999) in the Cohort of Eldorado Uranium Workers	16,236 Eldorado uranium male workers first employed in 1932–1980 and followed up through 1999.	Occupational exposure to radon decay products and gamma radiation.	Mean dose = 0.0522 Gy, range = <0.0234 – >0.1215 Gy.	Cerebrovascular disease mortality. ICD 9, codes not listed.	ERR/Gy (95% CI) = -0.29 (<–0.29, 0.27).
Kreuzer et al. (2013)	External Gamma Radiation and Mortality from Cardiovascular Diseases in the German WISMUT Uranium Miners Cohort Study, 1946-2008	58,982 German WISMUT cohort of uranium miners.	Occupational exposure to radon decay products and gamma radiation.	Mean dose = 0.047 Gy, range = 0.0002 – 0.909 Gy.	Cerebrovascular disease mortality. ICD 10 codes: 160-169.	ERR/Gy = 0.44 (95% CI: -0.16, 1.04).
Gillies et al. (2017)	Mortality from Circulatory Diseases and other Non-Cancer Outcomes among Nuclear Workers in France, the United Kingdom, and the United States (INWORKS)	308,297 nuclear workers exposed to low-dose radiation accumulated at low dose rates from France, United Kingdom and United States. International Nuclear Workers (INWORKS).	Low-level exposure to ionizing radiation at low dose rates.	Mean dose = 0.0252 Gy, range = 0 to 1.932 Gy.	Cerebrovascular disease mortality. ICD 9 codes: 430-438.	ERR/Gy = 0.50 (90% CI: 0.12, 0.94).
Bouet et al. (2019)	Analysis of the Association between Ionizing Radiation and Mortality in Uranium Workers from Five Plants Involved in the Nuclear Fuel Production Cycle in France	4,541 workers employed at least 6 months as members of the permanent staff in five plants involved in the nuclear fuel cycle in France between 1958 and 2006 and followed up between 1968 and 2013.	Chronic ionizing radiation exposure (both internal and external) with uranium bioassay results.	External mean dose = 0.011 Gy, range = 0 to 0.214 Gy.	Cerebrovascular disease mortality. ICD version 8 for period 1968–1978; ICD version 9 for 1979–1999 and ICD version 10 for 2000–2013. Specific codes not listed.	External dose: ERR/100 mGy = −0.03 (95% CI: NE, 6.14). No cerebrovascular data for internal doses.
Anderson et al. (2021)	Ischemic Heart and Cerebrovascular Disease Mortality in Uranium Enrichment Workers	23,731 male and 5,552 female US uranium enrichment workers from Oak Ridge Gaseous Diffusion Plant, Portsmouth Gaseous Diffusion Plant and Paducah Gaseous Diffusion Plant.	Occupational exposure to external ionizing radiation and internal exposure to uranium.	External mean dose = 0.044 Gy, range = 0 to 0.59 Gy. Cumulative absorbed lung dose of internal exposure to uranium mean dose = 0.001 Gy, range = 0 to 0.06 Gy.	Cerebrovascular disease mortality. ICD code versions used corresponded with the time of death.	External ERR/Gy = 0.49 (95% CI: −0.94, 2.5). Internal lung ERR/Gy = −0.13 (95% CI: −0.42 to 0.44).
Cha et al. (2020)*	Occupational Radiation Exposure and Morbidity of Circulatory Disease Among Diagnostic Medical Radiation Workers in South Korea	11,500 diagnostic medical radiation workers from South Korea.	Occupationally exposed radiologic technologists (majority X-ray radiation exposure) also radiologists, dentists, dental hygienists, nurses, physicians, and other medical assistants.	Mean cumulative heart dose for = 6.2 mGy, range = 0.002 to 72.9 mGy. Males had greater average dose than females (7.7 mGy vs. 2.7 mGy).	Cerebrovascular disease morbidity. ICD 10 codes: 160-169.	ERR/100 mGy = 3.10 (95% CI: -0.75 to 11.59) using morbidity data.
Boice et al. (2021)	Mortality Among Workers at the Los Alamos National Laboratory, 1943-2017	26,328 workers first employed between 1943 and 1980 at Los Alamos National Laboratory including contractors and followed through 2017.	Organ dose estimates for each worker considered all sources of Exposure, notably photons, neutrons, tritium, ^238^Pu and ^239^Pu.	Lung mean organ dose = 13.9 mGy, range = 0 to 1.25 Gy for a dose rating factor of 1.	Cerebrovascular disease mortality. ICD 9 codes: 430-438.	ERR/100mGy = 0.11 (95% CI: -0.35, 0.12).
Boice et al. (2021)	Mortality Among Medical Radiation Workers in the United States, 1965-2016	109,019 medical workers, 55,218 males (50.6%) and 53,801 females (49.4%). The medical worker cohort includes physicians and technologists (general radiology, interventional radiology/cardiology, nuclear medicine, and radiation oncology), nuclear pharmacists, medical and radiation physicists, nurses, veterinarians, chiropractors, dentists, and allied healthcare support workers monitored for radiation in similar environments.	Medical workers exposed to X-rays and gamma-rays with energies from about 0.02 MeV to slightly greater than 1 MeV.	The mean and median cumulative badge doses, i.e., H_P_(10) personal dose equivalents, were 63 mSv and 37 mSv, respectively. and ranged to over 500 mSv.	Cerebrovascular disease mortality. ICD 9 codes: 430-438.	ERR/100mGy = 0.04 (95% CI: -0.16, 0.23).
Hinksman et al. (2022)	Cerebrovascular Disease Mortality after Occupational Radiation Exposure Among the UK National Registry for Radiation Workers Cohort	166,812 nuclear workers from the UK National Registry for Radiation Workers (NRRW).	Occupational exposures commonly with X-ray and gamma-ray exposure with a small component from beta particles and neutrons.	Median dose = 3.1 mSV, range = 0 to 1.9 Sv. 94% of individuals received a total cumulative dose of less than 100 mSv.	Cerebrovascular disease mortality. ICD 9 codes: 4300-4389.	ERR/Sv = 0.57 (95% CI: 0.00, 1.31). Increased cerebrovascular disease mortality rates were observed after doses as low as 10–20 mSv.
**Chernobyl Catastrophe**						
Ivanov et al. (2006)*	The Risk of Radiation-induced Cerebrovascular Disease in Chernobyl Emergency Workers	61,017 Chernobyl emergency workers observed between 1986 and 2000 and within that group, 29,003 who arrived in the Chernobyl zone within the first year after the accident.	Exposure to radiation from the Chernobyl catastrophe.	Mean dose = 0.109 Gy, range = 0 to >0.5 Gy.	Cerebrovascular disease morbidity. ICD 10 codes: I60–I69.	ERR/Gy = 0.45 (95% CI: 0.11, 0.80) using morbidity data.
Kashcheev et al. (2016)*	Radiation-Epidemiological Study of Cerebrovascular Diseases in the Cohort of Russian Recovery Operation Workers of the Chernobyl Accident	53,772 Russian workers (liquidators) involved in recovery tasks after the Chernobyl accident who arrived in the zone of the Chernobyl accident within the first year after the accident (26 April 1986–26 April 1987).	Exposure to radiation from the Chernobyl catastrophe.	Mean dose = 0.161 Gy, range = 0.0001–1.24.	Cerebrovascular diseases morbidity. ICD 10 codes: 160 – 169.	ERR/Gy = 0.45 (95% CI: 0.28, 0.62) using morbidity data.
Loganovsky et al. (2020)	Radiation Risk Analysis of Neuropsychiatric Disorders in Ukrainian Chornobyl Catastrophe Liquidators	198 clean-up workers of the Chornobyl catastrophe (liquidators).	Exposure to radiation from the Chornobyl catastrophe including iodine-131.	Dose range 0.6–5900.0 mSv.	Acute cerebrovascular disorders (stroke) (ICD-9: 430.0–436.9; ICD-10: I60.0–I66.0). Chronic cerebrovascular disorders and sequelae of cerebrovascular disease (ICD-9: 438.0–439.9; ICD-10: I67, I69).	Relative risk related to internal controls: Acute cerebrovascular disorders (stroke): 1.40 (95% CI: 1.3,1.5). Chronic cerebrovascular disorders and sequelae of cerebrovascular disease: 1.23 (95% CI: 1.0, 1.5).
**Mayak Workers**						
Azizova et al. (2014)†	Cerebrovascular Diseases Incidence and Mortality in an Extended Mayak Worker Cohort 1948–1982	22,377 workers first employed at the Mayak Production Association (Mayak PA) in 1948–1982.	Occupational prolonged exposure to external gamma radiation and some with internal alpha radiation from deposited plutonium.	The mean total dose from external gamma rays were 0.54 ± 0.76 Gy (95% percentile 2.21 Gy) for males and 0.44 ± 0.65 Gy (95% percentile 1.87 Gy) for females. The mean plutonium body burden in the 31% of workers monitored for internal exposure was 1.32 ± 4.87 kBq (95% percentile 4.71 kBq) for males and 2.21 ± 13.24 kBq (95% percentile 4.56 kBq) for females. Mean total absorbed alpha-particles dose to the liver from incorporated plutonium was 0.23 ± 0.77 Gy (95% percentile 0.89 Gy) in males and 0.44 ± 2.11 Gy (95% percentile 1.25 Gy) in females.	Cerebrovascular disease morbidity and mortality. ICD 9 codes: 430-438.	External doses: ERR/Gy = 0.46 (95% CI: 0.37, 0.57) using morbidity data. Total absorbed dose to the liver from internal alpha-particle radiation exposure ERR/Gy = 0.28 (95% CI: 0.16, 0.42) using morbidity data. No significant ERR in cerebrovascular disease mortality.
Moseeva et al. (2014) ^†^	Risks of Circulatory Diseases among Mayak PA workers with Radiation Doses Estimated using the Improved Mayak Worker Dosimetry System 2008	18,856 men and women Mayak Nuclear workers.	Occupational prolonged exposure to external gamma radiation and some with internal alpha radiation from deposited plutonium.	Mean absorbed cumulative dose of 0.62 ± 0.80 Gy for males and 0.51 ± 0.68 Gy for females.	Cerebrovascular disease morbidity and mortality. ICD 9 codes: 430–438.	ERR/Gy = 0.511 (95% CI: 0.408, 0.614) using morbidity data. ERR/Gy = 0.057 (95% CI: -0.047, 0.161) using mortality data.
Azizova et al. (2018)	An Assessment of Radiation-Associated Risks of Mortality from Circulatory Disease in the Cohorts of Mayak and Sellafield Nuclear Workers	23,443 workers from the UK Sellafield Worker Cohort and Russian Mayak Worker Cohort with external gamma radiation and / or internal alpha radiation from deposited plutonium.	Occupational exposure to external gamma radiation and some with internal alpha radiation from deposited plutonium.	UK Sellafield cohort mean dose = 0.07 Gy, range = 0, 1.88 Gy. Mayak cohort mean dose = 0.52 Gy, range = 0, 8.4 Gy.	Cerebrovascular disease mortality. ICD-10 codes: 160-169.	UK Sellafield cohort ERR/Gy = 0.05 (95% CI: –0.46, 0.79). Mayak cohort ERR/Gy = 0.00 (95% CI: –0.06, 0.08).
Azizova (2022)	Mortality from Various Diseases of the Circulatory System in the Russian Mayak Nuclear Worker Cohort: 1948–2018	22,377 Russian nuclear workers of the Mayak Production Association (25.4% females) who were hired at the facility in 1948–1982 and followed up until end-2018 and a sub cohort of workers who were residents of the dormitory town of Ozyorsk.	Occupational prolonged exposure to external gamma radiation and some with internal alpha radiation from deposited plutonium.	External mean dose = 0.45 ± 0.65 Gy for males and mean dose = 0.37 ± 0.56 Gy for females. Internal mean dose = 0.18 ± 0.65 Gy for males and mean dose = 0.40 ± 1.92 Gy for females.	Cerebrovascular diseases mortality. ICD-9 codes: 430–438 including Ischemic stroke (ICD-9 code 434).	ERR/Gy = –0.02 (95% CI: –0.12, 0.11) associated with liver absorbed gamma ray dose. For the sub cohort of residents, ERR/Gy = 0.43 (95% CI: 0.08, 0.99) for mortality from ischemic stroke.
**Semipalatinsk Nuclear Test Study**						
Groche et al. (2011)	Mortality from Cardiovascular Diseases in the Semipalatinsk Historical Cohort, 1960–1999, and its Relationship to Radiation Exposure	19,545 persons of exposed to radioactive fallout from nuclear testing in the vicinity of the Semipalatinsk Nuclear Test Site and comparison villages in the Semipalatinsk region.	Fallout from nuclear testing at the Semipalatinsk Nuclear Test Site (SNTS), Kazakhstan.	Mean dose = 0.09 Gy, range = 0 to 0.63 Gy.	Cerebrovascular disease mortality ICD 9 codes: 430–438.	ERR/Gy = 2.96 (95% CI: 1.77, 4.14) for all settlements. ERR/Gy = 0.06 (95% CI: -0.65, 0.54) for exposed settlements. Significance explained by differences in background rates of stroke in exposed and unexposed cohorts.
**Diagnostically Exposed Groups**						
Tran et al. (2017)	Radiation-associated Circulatory Disease Mortality in a Pooled Analysis of 77,275 Patients from the Massachusetts and Canadian Tuberculosis Fluoroscopy Cohorts	77,275 total tuberculosis fluoroscopy patients (63,707 patients in Canada and 13,568 patients in Massachusetts).	X-ray fluoroscopy used in the course of treatment for tuberculosis.	Entire cohort: mean = 1.16 Gy, range = 0 - 27.77 Gy. Those with <0.5 Gy: mean = 0.18 Gy, range = 0- 0.50 Gy.	Cerebrovascular disease mortality. ICD 9 codes: 430-438.	ERR/Gy= -0.014 (95% CI: –0.067, 0.044) for entire cohort. ERR/Gy = 0.441 (95% CI: –0.119, 1.090) in those with doses less than 0.50 Gy.

^*^study includes analysis of morbidity, ^†^ study includes analyses of both mortality and morbidity.

Not all studies have consistently demonstrated an impact of low-to-moderate dose ionizing radiation on the risk of mortality from cerebrovascular disease, albeit some studies report a positive ERR value that is not significant. For example, in the Russian Mayak Worker Cohort, there was a significant positive ERR for cerebrovascular disease morbidity (ERR/Gy = 0.46, 95% CI: 0.37, 0.57), but not for mortality (Azizova et al., 2014; Moseeva et al., 2014). Follow up of the Mayak cohort suggest the most vulnerable to ischemic stroke morbidity were residents of the dormitory town of Ozyorsk (Azizova et al., 2022). Other reports of a positive but not significant ionizing radiation-induced ERR of mortality from cerebrovascular disease include the Sellafield Nuclear Workers from the United Kingdom (Azizova et al., 2018), nuclear industry workers from the 15 Country study (Vrijheid et al., 2007), German uranium miners (Kreuzer et al., 2013), persons exposed to radioactive fallout from nuclear testing in the vicinity of the Semipalatinsk Nuclear Test Site (Grosche et al., 2011), workers at the Los Alamos National Laboratory (Boice et al., 2021b), US uranium enrichment workers (Anderson et al., 2021), medical radiation workers in the United States (Boice et al., 2021a), medical radiation workers in South Korea (Cha et al., 2020) and tuberculosis fluoroscopy patients with doses less than 0.50 Gy (Tran et al., 2017). Yet, some studies suggest a negative ERR for cerebrovascular disease mortality, such as male Eldorado uranium workers (Lane et al., 2010) and uranium workers from plants involved in the French nuclear fuel production cycle (Bouet et al., 2019).

Collectively, these findings suggest that there may be a risk of cerebrovascular disease from terrestrial low-to-moderate dose ionizing radiation exposure, but it depends on multiple factors including time and dose of exposure, age of the cohort, baseline risk factors, assessment of cofounders including comorbid disease and socioeconomic status, and whether incidence/morbidity, or mortality of cerebrovascular disease is measured. It is important to note that many of these analyses use international classification of disease (ICD) codes that include stroke. However, these analyses do not usually separate by stroke type or do not specify. In combination with the preclinical evidence, these results suggest that ionizing radiation may produce vascular damage; however, how this may translate to vascular dysfunction in the brain and cerebrovascular disease morbidity and mortality in humans is still unclear.

### 3.8 Summary of the evidence base

**FIGURE 3:**
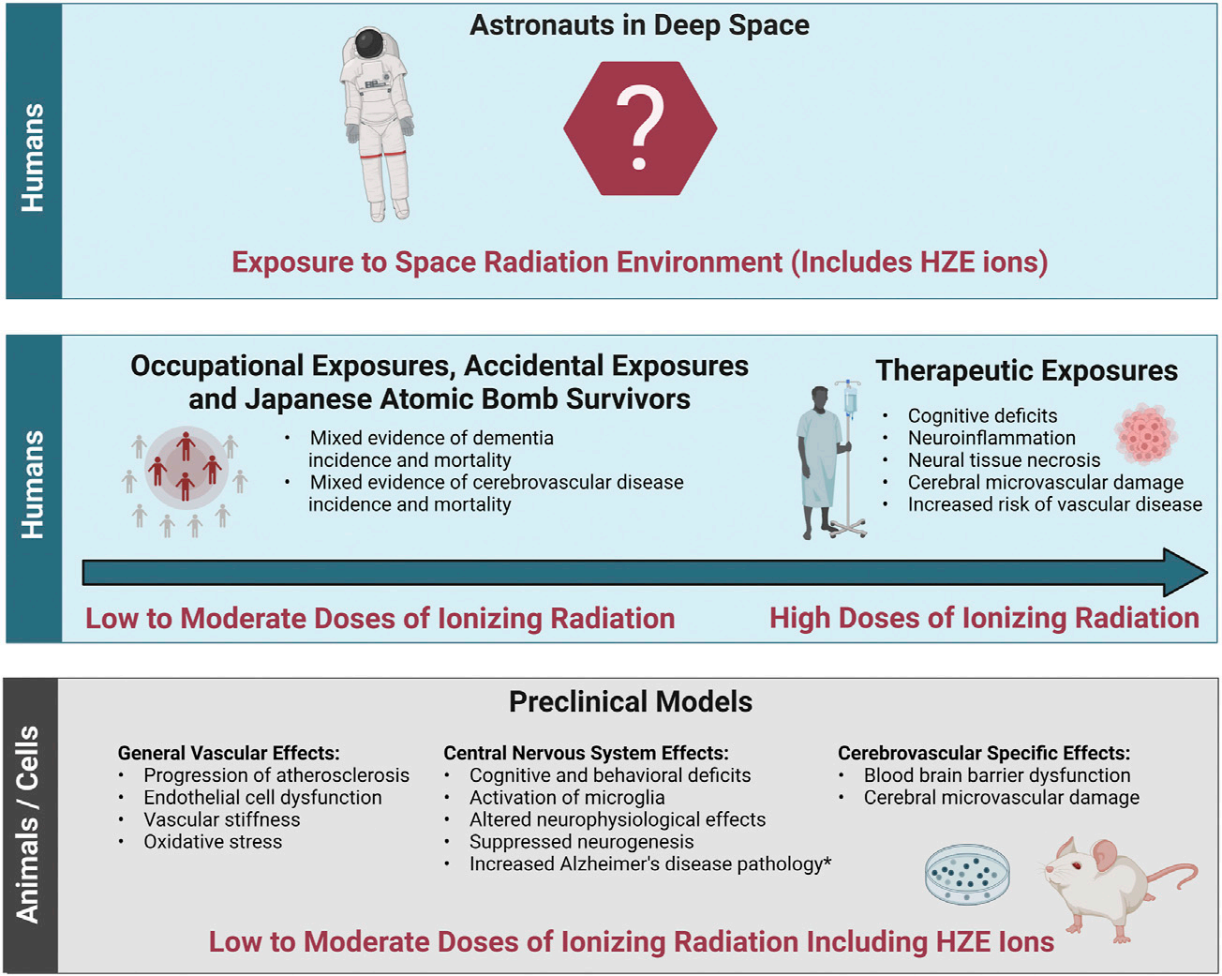
Evidence base of the effects of ionizing radiation relevant to cerebrovascular disease and dementia. The evidence base regarding the effects of ionizing radiation on cerebrovascular disease and dementia relevant to the space radiation environment consists of data from both humans and preclinical animal and cell models. Bottom panel: General vascular effects, central nervous system effects and cerebrovascular specific effects in preclinical animal and cell models irradiated with low (<0.1 Gy) to moderate (0.1 Gy–0.5 Gy) doses of protons or high (H) atomic number (Z) and energy (E) (HZE) ions. Middle panel left: Effects in cohorts of individuals exposed to low-to-moderate doses of terrestrial ionizing radiation including accidentally exposed individuals, Japanese Atomic Bomb Survivors, and occupationally exposed workers. Middle panel right: Effects in radiotherapy patients irradiated with high doses (ones to tens Gy and above). Top Panel: Translation of this evidence base into astronauts exposed to the space radiation environment is challenging due to the lack of evidence at the dose-rates and qualities related to long-term spaceflight such as deep space habitats and Mars missions. *Increased Alzheimer’s disease pathology has been demonstrated in some but not all transgenic mice with predisposition to develop Alzheimer’s disease. Image created with Biorender.com.

The evidence base describing the effects of ionizing radiation on the brain consists of therapeutic exposures, cohorts of people occupationally and accidentally exposed to low-to-moderate doses of terrestrial radiation, Japanese Atomic Bomb Survivors, and animal or cell models exposed to ionizing radiation including low-to-moderate doses of protons or HZE ions (Figure 3). Both human cohorts and experimental models show radiation-induced decrements to the CNS tissues with the potential for increased long-term risk for neurodegenerative diseases such as dementia. There is also epidemiological evidence for a potential ionizing radiation-induced risk of cerebrovascular disease morbidity and mortality, though the cohorts are heterogeneous, and the data is not conclusive. Experimental models evaluating the effects of ionizing radiation on the cerebrovascular system itself are limited, though a few studies have shown endothelial cell dysfunction leading to increased blood brain barrier permeability. In addition, ionizing radiation related general vascular damage from high-LET ions has been reported in the large central vessels like the aorta. Importantly, it is still unclear how decrements to the CNS may work independently or synergistically with decrements to the cerebral vessels. In addition, translation of terrestrial exposures and experimental models of cells and animals to humans in the space radiation environment is challenging.

## 4 Framework connecting cerebrovascular adverse outcomes and neural and cognitive adverse outcomes

**FIGURE 4:**
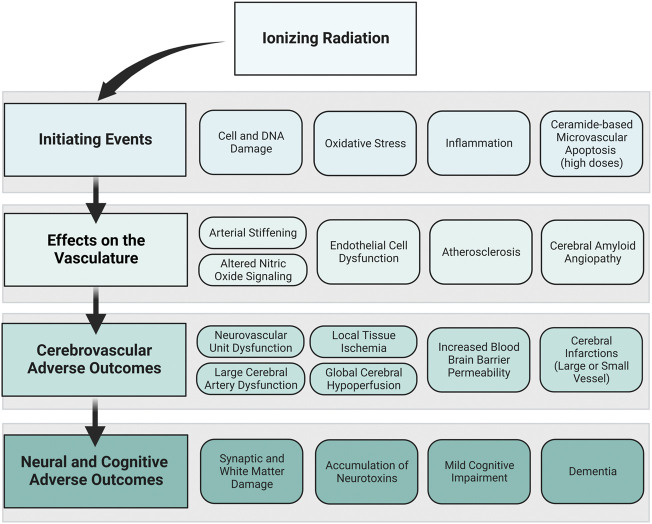
Proposed framework: ionizing radiation to neural and cognitive adverse outcomes *via* vascular mechanisms. How ionizing radiation may ultimately lead to neural and cognitive adverse outcomes through vascular mechanisms is displayed in this framework. The primary framework is displayed in the left column, where ionizing radiation creates initiating events that affect the vasculature, leading to cerebrovascular adverse outcomes and neural and cognitive adverse outcomes. To the right of each component of the primary framework, examples of individual events that may occur based on the data from the preclinical and human evidence base are shown. The order of boxes does not represent the order of events, and the time course and probability of these events are unknown. Image created with Biorender.com.

Developing a framework connecting cerebrovascular disease and neural and cognitive adverse outcomes offers a starting point for experimental and computational models to understand risk for space radiation-induced cerebrovascular and late neurocognitive effects. In our proposed framework, we show how initiating events caused by ionizing radiation exposure may affect the vascular cells of the brain and lead to cerebrovascular adverse outcomes and ultimately neural and cognitive adverse outcomes (Figure 4). The goal of the framework is not to suggest that cerebrovascular damage is a primary component for ionizing radiation-induced CNS injury, but rather to propose a mechanism by which cerebrovascular damage may lead to cognitive adverse outcomes in parallel or synergistic pathways with CNS tissue decrements. The time course and probability of the events are unknown, and the framework is exploratory and not meant to be definitive or absolute.

Ionizing radiation has been shown to directly affect both vascular and neural cells *via* damage to cell structure and DNA, increased oxidative stress and inflammation, and in situations with very high doses of ionizing radiation, it can cause ceramide-based apoptosis of microvascular cells. This may set off a cascade of events that elicits endothelial cell dysfunction, altered NO signaling, increased arterial stiffening, development of atherosclerosis and CAA. The myriad of insults to the vasculature may result in dysfunctional large and small cerebral blood vessels and lead to cerebrovascular adverse outcomes. For example, dysfunctional neurovascular units may cause a mismatch between cerebral metabolism and blood flow, which could result in both local tissue ischemia and global hypoperfusion. Insult to the cerebral endothelial cells may increase the permeability of the blood brain barrier causing the blood brain barrier to allow unwanted substances to cross into the brain parenchyma. In severe cases, cerebral infarctions could occur in either large or small vessels. These cerebrovascular adverse outcomes are related to neural and cognitive adverse outcomes. Ischemic or hypo-perfused tissue is vulnerable to synaptic and white matter damage and may lead to an accumulation of neurotoxins. Cerebrovascular damage may also reduce the ability of the blood vessels to detoxify or neutralize damaging constituents. Ultimately, irreversible damage to neural tissue and buildup of neurotoxic pathologies may result in cognitive impairment and neurodegenerative diseases including dementia. Dementia is an umbrella term that describes irreversible cognitive dysfunction with various causes including vascular dementia, Alzheimer’s disease, and Lewy body disease, though many dementia cases have mixed pathology.

Although the focus was on space radiation relevant exposures (i.e. high-LET exposures at low-to-moderate doses) the framework was developed with evidence from high-dose exposures in mind. It is possible that the framework could emphasize or de-emphasize certain mechanisms and outcomes depending on the exposure dose, dose-rate, duration of exposure, and radiation qualities (high verses low-LET). For example, ceramide-based microvascular apoptosis has been reported after high-dose exposures. However, it is unclear if this would occur at the doses relevant for long-duration space missions, which would de-emphasize its relevance for a space exposure scenario. Yet, compared with an acute high-dose exposure, the chronic nature of space radiation may increase probability of sustained oxidative damage, which would emphasize its relevance for a space exposure scenario. These are hypotheses that would need additional evidence to support.

The framework brings up multiple other questions for further research regarding its relevance for space exploration missions. For example, which parts of the framework are emphasized or de-emphasized based on LET? What about dose and dose-rate? What is the time-course by which adverse outcomes are apparent? I.e. is the timeline similar to what is observed in late-delayed radiation induced brain injury (6 months–1 year post exposure)? Is the timeline accelerated because of the chronic nature of the space radiation environment? Or is it decelerated because of the lower doses compared with most of the late-delated brain injury evidence base? Also, how much of the framework is influenced by the radiation exposure verses the individual exposed based on their age, sex, genotype and phenotype? There are still many unknowns regarding the impact of ionizing radiation on the cerebrovascular system, how vascular decrements may work in tandem or independently with decrements to the CNS tissues. More research is needed to describe how the evidence base translates to the space radiation environment.

## 5 Summary and conclusion

The purpose of this work was to review epidemiological and experimental evidence regarding the impact of ionizing radiation on cerebrovascular function and propose a framework by which ionizing radiation may lead to cerebrovascular, neural, and cognitive adverse outcomes. A review of epidemiological data from terrestrial ionizing radiation exposures and preclinical animal and cell models suggests that ionizing radiation may impact risk for cerebrovascular and neurodegenerative diseases. However, there are many gaps in the evidence base and differences between exposures and outcomes make it difficult to directly compare cohorts. Yet, multiple mechanisms were identified as potential targets by which ionizing radiation exposure may impact cerebrovascular function and consequent late neural and cognitive outcomes. These include initiating events such as increased oxidative stress and neuroinflammation, effects on the vasculature such as endothelial cell dysfunction and development of atherosclerosis, cerebrovascular adverse outcomes such as neurovascular unit dysfunction and increased blood brain barrier permeability, and neural and cognitive outcomes such as synaptic and white matter damage and cognitive impairment. The results of this work suggest that there are multiple potential mechanisms by which exposure to the space radiation environment could increase risk for late cerebrovascular diseases and dementia, however further research is necessary to understand if cerebrovascular adverse outcomes may promote neural, and cognitive adverse outcomes *via* parallel or synergistic pathways.

Understanding the impact of exposure to the space radiation environment is complex, as the space radiation environment consists of radiation exposures that are different in dose-rates and qualities from terrestrial exposures. The evidence base contains data from therapeutic exposures, cohorts of occupationally and accidentally exposed individuals, Japanese Atomic Bomb Survivors, and preclinical experimental models. In addition to the types of radiation exposure, there are other considerations to make when translating this evidence base into long-term neurodegenerative disease risk for astronauts exposed to the space radiation environment. For example, astronauts are healthier and may have higher “cognitive reserve” than the general population. A high cognitive reserve suggests that someone may be able to tolerate more age-related brain changes or pathology and still maintain function especially under stress and high workloads (Stern, 2009, 2012). In addition, sex differences in animal models exposed to HZE ions have been reported for both cognitive outcomes and structural plasticity of neurons (Krukowski et al., 2018a; Parihar et al., 2020). As men and women have different etiologies and outcomes of cerebrovascular and neurodegenerative diseases, sex-specific effects on risk will need to be considered (Appelros et al., 2009; Hanamsagar and Bilbo, 2016; Mielke, 2018). Age-related changes in cerebrovascular structure and function will also need to be considered, as cerebrovascular pathologies have been related to aging (Li et al., 2020). Finally, individual factors including genetics such as apolipoprotein E genotype, family history of disease, social determinants of health, and environmental factors can impact disease risk (Baumgart et al., 2015; Bellou et al., 2017).

In addition to ionizing radiation exposure, long-duration space travel includes multiple hazards including altered gravitational fields including microgravity, isolation and confinement/altered light-dark cycles, hostile and closed environment, and distance from Earth. Different hazards may have individual, and/or synergistic impacts on long-term health outcomes and could influence cerebrovascular outcomes and contribute to late neurodegenerative conditions. For example, increases in white matter hyperintensities and impaired cerebral drainage associated with microgravity have been reported in astronauts following spaceflight and are being investigated for potential impacts to crew behavior and performance (Alperin et al., 2017; Lee et al., 2019, 2021). In terrestrial cohorts these changes are associated with dementia risk (Weller et al., 2015; Alber et al., 2019). Likewise, a recent report of an internal jugular venous thrombus in an astronaut at the ISS spurred concern for altered coagulation states during spaceflight (Marshall-Goebel et al., 2019; Kim et al., 2021; Limper et al., 2021). The severity of risk for spaceflight induced venous thromboembolism and impaired cerebral drainage as well as the use of lower body negative pressure to mitigate risk are ongoing topics of debate (Harris et al., 2021). Furthermore, altered light-dark cycles may impact circadian rhythms as well as sleep duration and quality, also increasing potential risk for cerebrovascular disease (McDermott et al., 2018).

In summary, successful space exploration requires the characterization and management or mitigation of a variety of human health risks including late cerebrovascular and neurodegenerative diseases. Here we summarize the evidence base regarding the impact of ionizing radiation on the cerebrovascular system and propose a framework by which ionizing radiation could promote cerebrovascular and late cognitive adverse outcomes. As exposure to the space radiation environment has the potential to produce biological damage that is greater than terrestrial exposures, additional research that utilizes human data, preclinical models and computational models is needed to understand the long-term impact of human space exploration and occupation on brain health.

## Footnotes

1 This is not an endorsement by the National Aeronautics and Space Administration (NASA).

## Footnotes

1 This is not an endorsement by the National Aeronautics and Space Administration (NASA).
